# Does listening to the sound of yourself chewing increase your enjoyment of food?

**DOI:** 10.1186/1744-859X-5-22

**Published:** 2006-11-29

**Authors:** Kirsty E Amos, Shahram Anari, Charlotte A Buswell, Emma J McNeill, Athanasia Printza, Stephen J Ray, Isam Rustom

**Affiliations:** 1 Under the Auspices of the Master of Science in the Speech and Swallowing Research Course, University of Newcastle upon Tyne, UK

## Abstract

**Background:**

Anecdotal evidence suggests that listening to oneself eating results in a more pleasurable eating experience. Maximising the sensory experience of eating can result in increased oral intake and is potentially valuable in improving nutritional status in at-risk patients.

**Objective:**

This pilot study investigates the association between listening to the sound of oneself eating and the consequences on enjoyment of eating.

**Design:**

Prospective, randomized, controlled, cross-over trial of 10 fit, adult volunteers. Participants were timed eating a standardised amount of bread, and were randomized to eat in silence or whilst listening to their own amplified chewing and swallowing. Measurements of pulse and blood pressure were recorded throughout the procedure. Subjective pleasure scores were documented and the procedure repeated in the alternate study arm.

**Results:**

There was no significant relationship demonstrated between listening to oneself chewing and the enjoyment of eating.

**Conclusion:**

Although this small pilot study was unable to demonstrate a significant relationship between listening to oneself chewing and enjoyment of eating, other evidence suggests that distraction techniques have a beneficial effect on dietary intake. Such techniques can be applied in a clinical setting and further work in this area has valuable potential.

## Background

Managing inadequate dietary intake is prevalent throughout the health service, throughout multiple specialities, and in both community and hospital practice. Increased calorie intake can be achieved with dietary supplementation. There is also an important role for enhancing the enjoyment of eating[1].

Maximising the sensory experience of eating can result in increased intake. The use of flavour and odour enhancers to maximise dietary intake is well established. Schiffman found that older persons in long-term care facilities consume more food when flavour enhancement is used[1]. This results in improved functional and immunological status. Recent anecdotal evidence suggests that listening to oneself eating results in a more pleasurable eating experience[2]. Bellisle et al demonstrated that listening to background noise while eating results in increased intake[3]. The ability to positively encourage oral intake without recourse to medication would be extremely valuable for many client groups.

Research by Cuevas et al demonstrates an increase in swallowing rate in conditions of emotional arousal [4]. Bellisle also showed that chewing time is reduced if food is more palatable, and the experience of eating more pleasurable [5]. It would, therefore, seem logical that chewing and swallowing time could be used as a reflection of the palatability of food, and the pleasure of eating.

Differing and distinct cardiovascular responses can be seen following exposure to emotional stimuli [6]. In particular, pleasurable experiences are reflected by an increase in pulse and blood pressure without an overall increase in cardiac output[6]. Therefore, a pleasurable eating experience would be reflected by such changes in pulse and blood pressure measurements. If eating is more pleasurable as a result of listening to oneself chewing, chewing time would decrease and there would be a demonstrable cardiovascular response.

This study investigated whether listening to oneself chewing increases the pleasure of eating.

## Methods

### Subjects

Ten healthy volunteers were recruited from the students at the University of Newcastle-upon-Tyne (UK). Inclusion criteria included: consent to participate, and literacy sufficient to read instructions. Exclusion criteria were: hearing impairment, history of eating and drinking difficulties, food allergy, systemic medical problem (e.g. diabetes mellitus) or psychiatric disease.

### Design

Prospective, randomized, controlled, cross-over trial of 10 fit, adult volunteers. Pilot study.

### Procedure

Written informed consent was obtained. Height and weight were recorded in order to reflect the body mass index (BMI) of the participants. The BMI was estimated to exclude participants with eating disorders not acknowledged by them, since eating disorders influence the perception of eating pleasure.

Participants were block randomised to determine whether they would experience condition 'A' or condition 'B' first. Condition 'A' specified absence of amplified chewing sounds when the participants were chewing in a quiet room.Condition 'B' specified the condition when participants were listening to their chewing sound amplified and heard through the loud speakers placed half a metre in front of them.

To ensure consistent hunger for both conditions, participants did not eat or drink for two hours before each condition. This was monitored by participants indicating their hunger level on a Visual Analogue Scale (VAS) scaled from one to ten (with one indicating the least hunger) before starting to eat. Participants sat at a table and read an instruction sheet. The continuous cardiovascular monitor probe was placed on their non-dominant hand allowing them to feed themselves with their dominant hand. The diaphragm of the stethoscope was placed under the angle of the mandible by one researcher, and held throughout the procedure. The flat diaphragm was used for optimal amplification of the frequencies of both the chewing and the swallowing sounds. The loudspeaker was either on or off according to condition B or A respectively.

Participants ate one slice of bread at their own pace, and raised their hand to indicate when they perceived that they had swallowed all the bread and butter. One slice of white bread and butter (cut into four pieces) was used per participant in each condition. Systolic and diastolic blood pressure and also pulse were continuously measured during eating. Time taken to eat the bread was measured from when the leading edge of the first piece of bread passed the lips, to when the participant raised their hand. On completion of the first condition, participants were asked to rate their enjoyment of the eating experience on the visual analogue scale. Two hours later each participant then repeated the procedure under the other condition (A or B as above).

### Equipment

Equipment consisted of the following:

1) A personal computer [Toshiba notebook computer with Pentium processor (Intel, Santa Clara, CA, USA), a National Instruments (Austin, TX), DAQ pad 6020E (12 bit analogue to digital card), and Lab Microsoft Windows XP software]. 2) Pulse and blood pressure monitor [Finapres Datex Ohmeda 2300 (continuous cardiovascular monitor)]. 3) An amplifier [Chewing and swallowing sounds were amplified by a Littmann Cardio II stethoscope – St Paul, MN, USA – modified with two channel microphone amplifier FM317 and Yamaha MS101 monitor]. 4) A chronometer to measure the chewing time. 5) Scales and measuring tapes [Weight was measured using a domestic set of Salter scales and height by a tape fixed to a wall]. 6) Video camera [The experiment was recorded using a Panasonic M50 video camera.]

### Statistical analysis

The baseline physiologic parameters and the hunger VAS were compared between the two conditions using a paired t-test to ensure that no difference was present. In order to assess the change in the physiological parameters, the paired-sample t-test was used to compare the variables between the groups and within the same group. The pre-eating measurement was compared to the mean of the recording of each parameter for each participant during eating. The mean score was used as there was a continuous recording of the pulse and blood pressure through the Finapres machine. The variables assessed for comparison were:the mean/systolic/diastolic blood pressure, pulse rate, pre-eating hunger VAS and the post-eating pleasure VAS.

## Results

Ten participants were recruited for the study. The demographics of the study population are presented in Table 1. There was no significant difference between the baseline variables between the two conditions (Table 2). There was no significant change in the physiological parameters in either condition (Table 3). There was no statistically significant difference between conditions 'A' and 'B' in the time taken to eat or the VAS scoring of the pleasure of eating (p value 0.88 and 0.63 respectively). The power of this study was approximately 27% at the best (a retrospective power calculation using the systolic blood pressure data). A sample size of minimum 40 participants is needed to achieve a power of 80% with confidence level of 95%.

**Table 1:** Demographics of the study population (n: 10)

Female/Male	7/3
Age range (mean)	28–42 (33.4)
BMI* range (mean)	19–31 (26)

*BMI: Body Mass Index

**Table 2:** Comparison of the variables at baseline between the conditions.

	Condition 'A'	Condition 'B'	
Variables	Pre-test mean (SD)	Pre-test mean (SD)	p value

Systolic BP (mmHg)	137.8 (19.7)	140.0 (17.6)	0.82
Diastolic BP (mmHg)	105.1 (10.5)	107.1 (15.7)	0.76
Mean BP (mmHg)	118.1 (13.8)	120.5 (15.3)	0.76
Heart Rate (Beats/min)	75.6 (12.7)	70.2 (10.6)	0.38
VAS*- Hunger	60.7 (20.8)	54.1 (29.9)	0.42

*VAS: Visual Analogue Scale

**Table 3:** Measurement of variables before and during the test for each condition.

	Condition 'A'			Condition 'B'		
Variables	Pre-test Mean (SD)	During test Mean (SD)	p value	Pre-test Mean (SD)	During test Mean (SD)	p value

Systolic BP (mmHg)	137.8 (19.7)	136.0 (23.0)	0.85	140.0 (17.6)	128.1 (15.1)	0.17
Diastolic BP (mmHg)	105.1 (10.5)	111.7 (18.0)	0.39	107.1 (15.7)	106.4 (13.1)	0.91
Mean BP (mmHg)	118.1 (13.8)	123.8 (20.5)	0.52	120.5 (15.3)	117.8 (13.4)	0.68
Heart Rate (Beats/min)	75.6 (12.7)	78.0 (9.5)	0.50	70.2 (10.6)	76.9 (8.7)	0.14

Condition 'A': with no amplification of chewing sound; Condition 'B': with amplification of chewing sound.

## Discussion

In affluent societies eating is a pleasure and people do not eat merely to live. In addition to the physiological processes, eating behaviours are influenced by many factors -behavioural, social and environmental [7]. As a result, there are different methodologies applied to study different aspects of eating behaviours. A report on an actual 'experiment' of enhancing the enjoyment of eating by listening to the chewing sounds stimulated our interest [2]. We planned a study to evaluate a possible effect of listening to chewing sounds on the pleasure of eating.

The pleasure of eating is influenced by many parameters. In addition to satiation of one's hunger and the taste of the meal, certain social and environmental factors influence the pleasure. Although the complexity of the human brain makes it very difficult to apply general rules to the prediction of even straightforward behaviours such as eating, we tried to evaluate the level of pleasure by measuring changes in physiological parameters.

It has been shown that increased emotional arousal in healthy individuals is related to increased swallowing rate, as measured by the number of swallows per minute [4]. Bellisle et al showed that increased palatability of food increases the swallowing rate and reduces the chewing time [5]. Based on these reports we selected the duration of chewing as the outcome measure. Hetherington reports on studies showing that pleasure derived from eating a specific food is high at the beginning of a meal and stimulates further eating, but as eating progresses the participants report a decline in the pleasantness of the specific food [7]. By selecting a small test meal we attempted to prevent this effect from affecting our results.

There are discrete emotion-specific autonomic nervous system activities during periods of joy, sadness, fear, and anger. Sinha et al showed that the cardiovascular responses to joy were an increase of the heart rate and the systolic blood pressure without a significant increase of the diastolic blood pressure or the cardiac output [6]. In our study we continuously recorded the heart rate and the systolic, diastolic and mean blood pressure during the test period. Changes in these cardiovascular indices were the measure of increased enjoyment during eating.

Readiness to eat has been evaluated in different studies by recording blood glucose dynamics, by examining salivation and by self-reported hunger [7]. Self-report of hunger prior to test meals is a widely used tool in the research of eating behaviours. We used a Visual Analogue Scale of hunger. Meal eating patterns have been shown not to be sensitive to the level of food deprivation [5]. They were as responsive to sensory factors after an overnight fast, as after a usual breakfast to lunch interval. Therefore, our participants were expected to have almost equal levels of pre-test hunger in both phases of the study.

A pleasure VAS was considered important to elicit a direct response to the meal by participants. It is difficult to distinguish between measures of hunger, appetite and enjoyment of eating [7].

The choice of test meal is very important in the research in eating behaviour in humans. Quantities, types and textures vary and their selection depends on the research question [5,7]. In our study, a slice of bread was selected as it is generally considered palatable, is a small meal, requires chewing and is easy to chew regardless of participant's dental status. Studies have shown that meals interrupted for comments on palatability last longer and involve greater quantities of food [7]. In this study, participants were instructed to eat the whole slice of bread without interruption for comments or drinking. Test meals consisting of different food consistencies, producing more chewing sounds should be considered in future studies.

Other methodological considerations to take into account are that the artificial conditions of a laboratory setting may affect eating behaviours. During test meals, three researchers were present in the room with the participant. Being observed while eating may affect eating behaviour. These are important limitations of any eating behaviour study. It must be noted that the participants had visual, tactile and olfactory access to the food item in addition to the auditory effect. It would be interesting to exclude other sensory inputs while evaluating the potential enhancement of eating pleasure by listening to chewing sounds.

In this study, there was no significant difference in the pleasure scores and in the time taken to chew and swallow one slice of bread between two different test conditions. There were also no significant changes of cardiovascular indices between the two meal conditions. Although an order effect was not anticipated in this study participants were randomly assigned to either have the meal with or without listening to the chewing sounds first.

The magnitude of the intervention effect may have been insufficient to produce a measurable result. There is no evidence regarding the sound pressure level generated by a loudspeaker that would be considered as pleasant and sufficient to produce an effect. The room was quiet but not sound-treated, as a result there was some background noise and noise from the recording equipment. Cervical auscultation for dysphagia is usually performed at the lateral aspect of the neck [8,9]. In this study a modified stethoscope was located immediately below the angle of the jaw.

The magnitude of stimulation (eating and/or enjoyment) necessary to produce cardiovascular reactions detectable through blood pressure and pulse changes is not known. Changes in blood pressure and pulse rate in other studies were elicited with a methodology that involved stronger stimulation over longer periods [6]. The effect of controlled experimental conditions in laboratory settings on the pleasure of eating may be greater than the effect of the experimental intervention. This is a limitation of these types of studies.

To our knowledge, this is the first study to assess the effect of listening to chewing sounds on the pleasure received from eating. This pilot study did not show a relationship between listening to oneself chewing and enjoyment of eating. One reason may be the fact that the study population was small and so failed to generate sufficient power for analysis. A further study with a greater number of participants may identify some trends of influence.
